# 1-Bromo­methyl-4-aza-1-azoniabicyclo­[2.2.2]octane bromide

**DOI:** 10.1107/S1600536810000292

**Published:** 2010-01-16

**Authors:** Aaron D. Finke, Danielle L. Gray, Jeffrey S. Moore

**Affiliations:** a University of Illinois, School of Chemical Sciences, Box 77-5, 600 South Mathews Avenue, Urbana, Illinois 61801, USA; b University of Illinois, School of Chemical Sciences, Box 59-1, 505 South Mathews Avenue, Urbana, Illinois 61801, USA; c University of Illinois, School of Chemical Sciences, Box 55-5, 600 South Mathews Avenue, Urbana, Illinois 61801, USA

## Abstract

The title compound, C_7_H_14_BrN_2_^+^·Br^−^, was prepared by nucleophilic substitution of DABCO (systematic name: 1,4-diaza­bicyclo­[2.2.2]octa­ne) with dibromo­methane in acetone. The structure features Br⋯H close contacts (2.79 and 2.90 Å) as well as a weak bromine–bromide inter­action [3.6625 (6) Å].

## Related literature

For use of DABCO as an organocatalyst, see Basaviah *et al.* (2003 ▶). For related haloalkyl­ations of DABCO, see: Almarzoqi *et al.* (1986 ▶); Fronczek *et al.* (1990 ▶); Gustafsson *et al.* (2005 ▶); Banks *et al.* (1993 ▶); Batsanov *et al.* (2005 ▶); Fletcher Claville *et al.* (2007 ▶). For inversion twinning, see: Flack & Bernardinelli (2000 ▶).
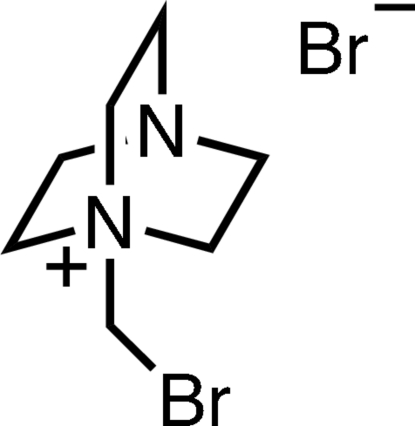


## Experimental

### 

#### Crystal data

C_7_H_14_BrN_2_^+^·Br^−^
*M* *_r_* = 286.02
Orthorhombic, 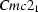

*a* = 7.1100 (3) Å
*b* = 11.8085 (5) Å
*c* = 11.7702 (5) Å
*V* = 988.21 (7) Å^3^
*Z* = 4
Mo *K*α radiation
μ = 8.15 mm^−1^
*T* = 193 K
0.36 × 0.35 × 0.06 mm

#### Data collection

Bruker Kappa APEXII CCD diffractometer
Absorption correction: integration [*SHELXTL* (Sheldrick, 2008 ▶) and *XPREP* (Bruker, 2005 ▶)] *T*_min_ = 0.151, *T*_max_ = 0.744
7347 measured reflections
991 independent reflections
954 reflections with *I* > 2σ(*I*)
*R*_int_ = 0.050

#### Refinement

*R*[*F* ^2^ > 2σ(*F* ^2^)] = 0.022
*wR*(*F* ^2^) = 0.052
*S* = 1.10
991 reflections
61 parameters
1 restraint
H-atom parameters constrained
Δρ_max_ = 0.42 e Å^−3^
Δρ_min_ = −0.44 e Å^−3^
Absolute structure: Flack (1983 ▶), 468 Friedel pairs
Flack parameter: −0.004 (17)

### 

Data collection: *APEX2* (Bruker, 2004 ▶); cell refinement: *SAINT* (Bruker, 2005 ▶); data reduction: *SAINT* and *XPREP* (Bruker, 2005 ▶); program(s) used to solve structure: *SHELXS97* (Sheldrick, 2008 ▶); program(s) used to refine structure: *SHELXL97* (Sheldrick, 2008 ▶); molecular graphics: *SHELXTL* (Sheldrick, 2008 ▶) and *CrystalMaker* (CrystalMaker, 1994 ▶); software used to prepare material for publication: *XCIF* (Bruker, 2005 ▶).

## Supplementary Material

Crystal structure: contains datablocks I, global. DOI: 10.1107/S1600536810000292/pk2223sup1.cif

Structure factors: contains datablocks I. DOI: 10.1107/S1600536810000292/pk2223Isup2.hkl

Additional supplementary materials:  crystallographic information; 3D view; checkCIF report

## Figures and Tables

**Fig. 1.**
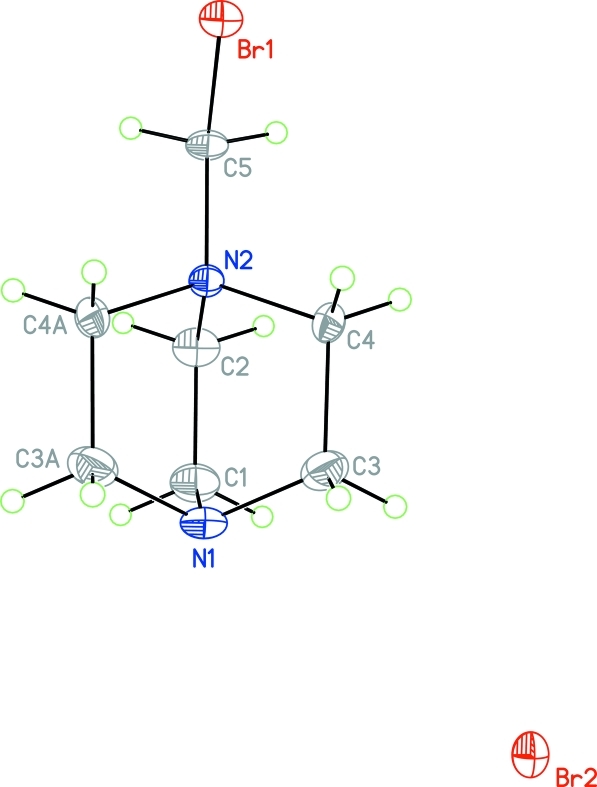
Thermal ellipsoid plot showing non-H atoms at 35% probability and H atoms as arbitrary small spheres. The atoms labeled A are related by the symmetry operator (1 - x, y, z).

**Fig. 2.**
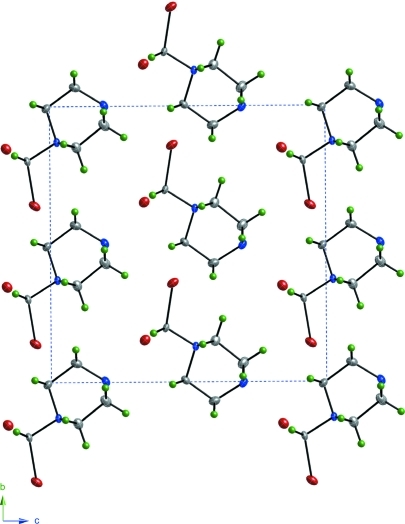
A packing plot of the unit cell as viewed down the a-axis showing 35% probability ellipsoids for non-H atoms. H atoms have been removed to improve clarity.

## supplementary crystallographic information

### Comment

The nucleophilicity of 1,4-diazabicyclo[2.2.2]octane (DABCO) has enabled it to be an excellent organocatalyst for a variety of reactions, in particular the Baylis-Hillman reaction (Basaviah *et al.*, 2003). Furthermore, DABCO can undergo substitution with even relatively unreactive electrophiles such as dichloromethane (Almarzoqi *et al.*, 1986).

We have isolated crystals of the title compound of sufficient quality for crystallographic analysis.  Typically, the reaction between dibromomethane and DABCO proceeds quickly in acetone, resulting in the immediate precipitation of the monoalkylated bromide salt that is insoluble in acetone.  However, at sufficiently low concentrations of reactants, slow crystallization of the product occurs.

The title molecule crystallizes in a non-centrosymmetric space group, Cmc2_1_.  One notable feature of this structure is a close contact between free bromide and the bromomethyl hydrogen (Br2···H5A) of 2.794 (3) Å. Another close contact for H4B···Br2 (2.899 (3) Å) was found. There is also a relatively close contact between the covalently-bound bromine and bromide anion (Br1···Br2 3.6625 (6) Å).  However, this distance is significantly longer than the Br···Br interaction seen in a related structure, (bromomethyl)trimethylammonium bromide (Br···Br = 3.369 Å) (Fletcher Claville *et al.* 2007).

### Experimental

Dibromomethane (10 mmol) was added to a solution of DABCO (10 mmol) in acetone (100 ml). Colorless plates of poor quality evolve almost immediately, which after 1 h are filtered. The filtrate is sealed in a flask and left to sit for 1 week, after which prisms suitable for X-ray analysis form on the side of the flask.

### Refinement

A structural model consisting of one symmetrically independent molecule was developed. All non-hydrogen atoms were refined with anisotropic displacement parameters. All hydrogen atoms were placed in ideal positions and refined as riding atoms. The ideally calculated H atoms on C1, C2, and C5 are related by the symmetry operation (1 - *x*, *y*, *z*). For these H atoms the special position constraints were suppressed to allow for the correct calculation of the idealized H atom positions. For all H atoms the U_iso_ values were assigned as 1.2 times the carrier *U*_eq_.  On the basis of 468 unmerged Friedel opposites, the likelihood of inversion twinning is negligible (Flack, 1983; Flack& Bernardinelli, 2000).

### Crystal data

**Table:**

C_7_H_14_BrN_2_^+^·Br^−^	*F*(000) = 560
*M**_r_* = 286.02	*D*_x_ = 1.922 Mg m^−^^3^
Orthorhombic, *C**m**c*2_1_	Mo *K*α radiation, λ = 0.71073 Å
Hall symbol: C 2c -2	Cell parameters from 3160 reflections
*a* = 7.1100 (3) Å	θ = 3.3–26.3°
*b* = 11.8085 (5) Å	µ = 8.15 mm^−^^1^
*c* = 11.7702 (5) Å	*T* = 193 K
*V* = 988.21 (7) Å^3^	Plate, colourless
*Z* = 4	0.36 × 0.35 × 0.06 mm

### Data collection

**Table:**

Bruker Kappa APEXII CCD diffractometer	991 independent reflections
Radiation source: fine-focus sealed tube	954 reflections with *I* > 2σ(*I*)
graphite	*R*_int_ = 0.050
φ and ω scans	θ_max_ = 25.4°, θ_min_ = 3.3°
Absorption correction: integration (*SHELXTL* and *XPREP*; Bruker, 2005)	*h* = −8→8
*T*_min_ = 0.151, *T*_max_ = 0.744	*k* = −14→14
7347 measured reflections	*l* = −14→14

### Refinement

**Table:**

Refinement on *F*^2^	Secondary atom site location: difference Fourier map
Least-squares matrix: full	Hydrogen site location: inferred from neighbouring sites
*R*[*F*^2^ > 2σ(*F*^2^)] = 0.022	H-atom parameters constrained
*wR*(*F*^2^) = 0.052	*w* = 1/[σ^2^(*F*_o_^2^) + (0.0248*P*)^2^] where *P* = (*F*_o_^2^ + 2*F*_c_^2^)/3
*S* = 1.10	(Δ/σ)_max_ < 0.001
991 reflections	Δρ_max_ = 0.42 e Å^−^^3^
61 parameters	Δρ_min_ = −0.44 e Å^−^^3^
1 restraint	Absolute structure: Flack (1983), 468 Friedel pairs
Primary atom site location: structure-invariant direct methods	Flack parameter: −0.004 (17)

### Special details

**Table:**

Experimental. One distinct cell was identified using *APEX2* (Bruker, 2004). Seven frame series were integrated and filtered for statistical outliers using *SAINT* (Bruker, 2005) then corrected for absorption by integration using *SHELXTL*/*XPREP* V2005/2 (Bruker, 2005) before using *SAINT*/*SADABS* (Bruker, 2005) to sort, merge, and scale the combined data. No decay correction was applied.
Geometry. All e.s.d.'s (except the e.s.d. in the dihedral angle between two l.s. planes) are estimated using the full covariance matrix. The cell e.s.d.'s are taken into account individually in the estimation of e.s.d.'s in distances, angles and torsion angles; correlations between e.s.d.'s in cell parameters are only used when they are defined by crystal symmetry. An approximate (isotropic) treatment of cell e.s.d.'s is used for estimating e.s.d.'s involving l.s. planes.
Refinement. Structure was phased by direct methods (Sheldrick, 2008). Systematic conditions suggested the ambiguous space group. The space group choice was confirmed by successful convergence of the full-matrix least-squares refinement on *F*^2^. The highest peaks in the final difference Fourier map were in the vicinity of atoms Br1 and Br2; the final map had no other significant features. A final analysis of variance between observed and calculated structure factors showed some dependence on amplitude and resolution.

### Fractional atomic coordinates and isotropic or equivalent isotropic displacement parameters (Å^2^)

**Table:**

	*x*	*y*	*z*	*U*_iso_*/*U*_eq_	Occ. (<1)
Br1	0.5000	0.14054 (4)	−0.05638 (4)	0.03174 (19)	
Br2	0.0000	0.65245 (4)	0.34152 (3)	0.02920 (17)	
N1	0.5000	0.5040 (4)	0.1977 (4)	0.0281 (9)	
N2	0.5000	0.3707 (3)	0.0253 (3)	0.0188 (8)	
C1	0.5000	0.5692 (5)	0.0917 (5)	0.0360 (13)	
H1A	0.6126	0.6185	0.0898	0.043*	0.50
H1B	0.3874	0.6185	0.0898	0.043*	0.50
C2	0.5000	0.4932 (4)	−0.0128 (4)	0.0291 (12)	
H2A	0.3871	0.5086	−0.0595	0.035*	0.50
H2B	0.6129	0.5086	−0.0595	0.035*	0.50
C3	0.3326 (5)	0.4320 (3)	0.1979 (3)	0.0369 (9)	
H3A	0.2189	0.4804	0.1960	0.044*	
H3B	0.3294	0.3875	0.2691	0.044*	
C4	0.3283 (5)	0.3507 (3)	0.0964 (3)	0.0264 (8)	
H4A	0.3263	0.2714	0.1236	0.032*	
H4B	0.2137	0.3640	0.0506	0.032*	
C5	0.5000	0.3026 (4)	−0.0825 (4)	0.0234 (10)	
H5A	0.6125	0.3229	−0.1278	0.028*	0.50
H5B	0.3875	0.3229	−0.1278	0.028*	0.50

### Atomic displacement parameters (Å^2^)

**Table:**

	*U*^11^	*U*^22^	*U*^33^	*U*^12^	*U*^13^	*U*^23^
Br1	0.0285 (4)	0.0282 (3)	0.0385 (4)	0.000	0.000	−0.0120 (3)
Br2	0.0230 (3)	0.0325 (3)	0.0321 (4)	0.000	0.000	0.0042 (3)
N1	0.037 (2)	0.027 (2)	0.021 (2)	0.000	0.000	−0.0071 (18)
N2	0.021 (2)	0.023 (2)	0.013 (2)	0.000	0.000	−0.0006 (16)
C1	0.046 (3)	0.024 (3)	0.038 (3)	0.000	0.000	−0.005 (2)
C2	0.042 (3)	0.023 (3)	0.022 (3)	0.000	0.000	0.006 (2)
C3	0.039 (2)	0.037 (2)	0.034 (2)	−0.0058 (18)	0.0135 (16)	−0.0103 (18)
C4	0.0189 (18)	0.035 (2)	0.0254 (17)	−0.0029 (15)	0.0062 (12)	−0.0040 (13)
C5	0.032 (3)	0.025 (2)	0.013 (2)	0.000	0.000	−0.0048 (18)

### Geometric parameters (Å, °)

**Table:**

Br1—C5	1.938 (5)	C3—C4	1.532 (4)
N1—C3^i^	1.463 (4)	C3—H3A	0.9900
N1—C3	1.464 (4)	C3—H3B	0.9900
N1—C1	1.466 (6)	C4—H4A	0.9900
N2—C4	1.499 (4)	C4—H4B	0.9900
N2—C4^i^	1.499 (4)	C5—H5A	0.9900
N2—C5	1.502 (6)	C5—H5B	0.9900
N2—C2	1.514 (6)	Br1—Br2^ii^	3.6625 (6)
C1—C2	1.522 (7)	Br2—H5A^iii^	2.79
C1—H1A	0.9900	Br2—H5B^iv^	2.79
C1—H1B	0.9900	Br2—H4B^v^	2.90
C2—H2A	0.9900	Br2—H4B^iv^	2.90
C2—H2B	0.9900		
			
C3^i^—N1—C3	108.9 (4)	H2A—C2—H2B	108.3
C3^i^—N1—C1	107.8 (3)	N1—C3—C4	112.3 (3)
C3—N1—C1	107.8 (3)	N1—C3—H3A	109.1
C4—N2—C4^i^	109.1 (4)	C4—C3—H3A	109.1
C4—N2—C5	112.8 (2)	N1—C3—H3B	109.1
C4^i^—N2—C5	112.8 (2)	C4—C3—H3B	109.1
C4—N2—C2	108.4 (2)	H3A—C3—H3B	107.9
C4^i^—N2—C2	108.4 (2)	N2—C4—C3	108.7 (3)
C5—N2—C2	105.2 (3)	N2—C4—H4A	109.9
N1—C1—C2	112.2 (4)	C3—C4—H4A	109.9
N1—C1—H1A	109.2	N2—C4—H4B	109.9
C2—C1—H1A	109.2	C3—C4—H4B	109.9
N1—C1—H1B	109.2	H4A—C4—H4B	108.3
C2—C1—H1B	109.2	N2—C5—Br1	113.2 (3)
H1A—C1—H1B	107.9	N2—C5—H5A	108.9
N2—C2—C1	108.9 (4)	Br1—C5—H5A	108.9
N2—C2—H2A	109.9	N2—C5—H5B	108.9
C1—C2—H2A	109.9	Br1—C5—H5B	108.9
N2—C2—H2B	109.9	H5A—C5—H5B	107.7
C1—C2—H2B	109.9		
			
C3^i^—N1—C1—C2	58.7 (2)	C4^i^—N2—C4—C3	59.3 (4)
C3—N1—C1—C2	−58.7 (2)	C5—N2—C4—C3	−174.6 (3)
C4—N2—C2—C1	59.1 (2)	C2—N2—C4—C3	−58.6 (3)
C4^i^—N2—C2—C1	−59.1 (2)	N1—C3—C4—N2	−0.5 (4)
C5—N2—C2—C1	180.0	C4—N2—C5—Br1	−62.1 (2)
N1—C1—C2—N2	0.0	C4^i^—N2—C5—Br1	62.1 (2)
C3^i^—N1—C3—C4	−57.5 (5)	C2—N2—C5—Br1	180.0
C1—N1—C3—C4	59.2 (4)		

Symmetry codes: (i) −*x*+1, *y*, *z*; (ii) −*x*+1/2, −*y*+1/2, *z*−1/2; (iii) −*x*+1, −*y*+1, *z*+1/2; (iv) −*x*, −*y*+1, *z*+1/2; (v) *x*, −*y*+1, *z*+1/2.
